# Lack of catch-up in weight gain may intermediate between pregnancies with hyperemesis gravidarum and reduced fetal growth: the Japan Environment and Children’s Study

**DOI:** 10.1186/s12884-022-04542-0

**Published:** 2022-03-12

**Authors:** Naho Morisaki, Chie Nagata, Seiichi Morokuma, Kazushige Nakahara, Kiyoko Kato, Masafumi Sanefuji, Eiji Shibata, Mayumi Tsuji, Masayuki Shimono, Toshihiro Kawamoto, Shouichi Ohga, Koichi Kusuhara, Japan Environment, Children’s Study Group, Hirohisa Saito, Reiko Kishi, Nobuo Yaegashi, Koichi Hashimoto, Chisato Mori, Shuichi Ito, Zentaro Yamagata, Hidekuni Inadera, Michihiro Kamijima, Toshio Heike, Hiroyasu Iso, Masayuki Shima, Yasuaki Kawai, Narufumi Suganuma, Koichi Kusuhara, Takahiko Katoh

**Affiliations:** 1 Department of Social Medicine, National Center for Child Health and Development, Setagaya, Tokyo, Japan; 2 Center for Postgraduate Education and Training, National Center for Child Health and Development, Setagaya, Tokyo, Japan; 3 Department of Health Sciences, Graduate School of Medical Sciences, Kyushu University, 3-1-1 Maidashi, Higashi-ku, Fukuoka, Fukuoka 812-8582 Japan; 4 Department of Obstetrics and Gynecology, Graduate School of Medical Sciences, Kyushu University, Fukuoka, Fukuoka Japan; 5 Department of Pediatrics, Graduate School of Medical Sciences, Kyushu University, Fukuoka, Fukuoka Japan; 6 Research Center for Environment and Developmental Medical Sciences, Kyushu University, Fukuoka Fukuoka, Japan; 7 Department of Obstetrics and Gynecology, School of Medicine, University of Occupational and Environmental Health, Kitakyushu, Fukuoka Japan; 8 Japan Environment and Children’s Study, UOEH Subunit Center, University of Occupational and Environmental Health, Kitakyushu, Fukuoka Japan; 9 Department of Environmental Health, School of Medicine, University of Occupational and Environmental Health, Kitakyushu, Fukuoka Japan; 10 Department of Pediatrics, School of Medicine, University of Occupational and Environmental Health, Kitakyushu, Fukuoka Japan

**Keywords:** Hyperemesis gravidarum, Nausea and vomiting of pregnancy, Morning sickness, Gestational weight gain, Fetal growth, Birth weight, Small for gestational age

## Abstract

**Background:**

Women with nausea and vomiting of pregnancy (NVP) have higher birth weight infants, while those with hyperemesis gravidarum, a severe manifestation of NVP, have lower birth weight infants. We aimed to investigate the associations between maternal weight loss (a consequence of hyperemesis gravidarum), NVP, and infant birth weight.

**Methods:**

This study was a secondary analysis of a nationwide birth cohort in Japan. Singleton pregnancies delivered at 28–41 weeks of gestation were included in the analysis. Women were categorized based on their weight change in the 1^st^ trimester (as a proportion to their pre-pregnancy weight: >  + 3%, > 0 to + 3%, > -3 to 0%, > -5 to -3%, ≤ -5%) and severity of NVP (no nausea, only nausea, vomiting but able to eat, vomiting and unable to eat). The effects of weight change and severity of NVP on infant birth weight and small for gestational age (SGA) were assessed using regression models. We further examined how these effects could be modified by maternal weight gain up to the 2^nd^ trimester.

**Results:**

Among 91,313 women, 5,196 (5.7%) lost ≥ 5% of their pre-pregnancy weight and 9,983 (10.9%) experienced vomiting and were unable to eat in the 1^st^ trimester. Women with weight loss ≥ 5% in the 1^st^ trimester had infants 66 (95% CI: 53, 78) g lighter and higher odds of SGA (aOR: 1.29; 95% CI: 1.14, 1.47) than women who gained > 3% during the same period. However, when adjusting for weight gain up to the 2^nd^ trimester, women with weight loss ≥ 5% in the 1^st^ trimester had infants 150 (95% CI: 135, 165) g heavier and lower odds of SGA (aOR: 0.39; 95% CI: 0.33, 0.46) than those who gained > 3% during the same period. In contrast, women with more severe NVP tended to have infants with larger birth weight and lower odds of SGA compared to women without NVP. These trends were strengthened when adjusting for weight gain up to the 2^nd^ trimester.

**Conclusions:**

Our study suggests the possibility that reduced fetal growth in pregnancies with hyperemesis gravidarum may be caused by the lack of catch-up in gestational weight gain up to the 2^nd^ trimester.

**Supplementary Information:**

The online version contains supplementary material available at 10.1186/s12884-022-04542-0.

## Background

Nausea and vomiting of pregnancy (NVP), commonly experienced by 35–91% of pregnant women typically in the 1^st^ trimester, is characterized by nausea and vomiting [1–4]. Hyperemesis gravidarum is a severe manifestation of NVP and is described as involving severe nausea and vomiting, which may lead to a loss of more than 5% of the pregnant woman’s pre-pregnancy weight, dehydration, electrolyte imbalances, and antenatal hospital admission [5–7]. This condition reportedly affects 0.3%-3.6% of pregnant women [2, 8–10].

The potential adverse effects of hyperemesis gravidarum on fetal growth have been reported, and a systematic review and large population-based study have confirmed this association [11, 12]. However, another meta-analysis and large observational study also showed that women who experienced NVP gave birth to heavier infants than those who did not [13, 14]. Given that hyperemesis gravidarum is a severe manifestation of NVP, the apparently contradictory association with fetal growth invites inquiry. There are also studies that have focused on the adverse impact of insufficient gestational weight gain [15–17] and weight gain in early pregnancy [18, 19] on fetal growth. The lack of definite diagnostic criteria for hyperemesis gravidarum may have confused the situation and made research synthesis challenging [6, 7]. Nonetheless, few studies have investigated the mechanism underlying this paradoxical phenomenon.

In this study, we focused on “maternal weight change”, as maternal weight loss is one of the major consequences of hyperemesis gravidarum. We aimed to investigate 1) the association between maternal weight change in the 1^st^ trimester and birth weight of infants, 2) the association between the severity of NVP symptoms and birth weight of infants; and 3) how these associations change after adjusting for differences in maternal weight gain up to the 2^nd^ trimester.

## Methods

### Study design, setting, and study sample

We used data collected from the Japan Environment and Children’s Study (JECS), a nationwide prospective cohort study of pregnant women, their spouses, and their children in Japan. The Review Board on Epidemiological Studies of the Ministry of the Environment and the ethics committees of all participating institutions approved the JECS protocol. Written informed consent was obtained from all participants. The study was conducted in accordance with the relevant national and institutional guidelines as well as the Declaration of Helsinki.

The detailed methodology of this cohort has been previously reported [20, 21]. In brief, pregnant women were recruited through 1) the first antenatal visit at participating health care institutions, and 2) the local government offices issuing the Mother–Child Health Handbook from January 2011 to March 2014 in 15 study regions throughout Japan. During pregnancy, participating women were asked to fill out two questionnaires: one administered at recruitment and another administered at mid-pregnancy, which captured their demographics, lifestyle, behaviors, and medical history. Birth characteristics and medical information were transcribed separately from medical records.

**Fig. 1:**
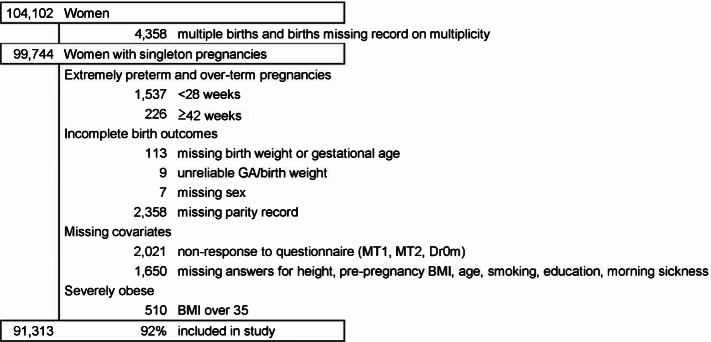
Population flow chart. Legends: BMI, body mass index; GA, gestational age

In total, 104,102 births were born among the recruited women. For this study, we used the dataset of the birth characteristics “jecs-ag-20160424” which was created in April 2016 and revised in October 2016. Among 99,744 singleton pregnancies in the study, we excluded miscarriages and births (including stillbirths) before 28 weeks (*n* = 1,537), post-term births more than or equal to 42 weeks (*n* = 226), and births with missing background characteristics (*n* = 6,158). We also excluded births from severely obese women with body mass index (BMI) over 35 (*n* = 510) because they were considered as outliers of the study sample (+ 4.18 standard deviation) and may include unreliable measurements/records. Thus, we based our analysis on 91,313 (92%) subjects (Fig. 1).

### Variable definitions

Our primary variable of interest was maternal weight change during the 1^st^ trimester. Weight change in the 1^st^ trimester, as well as weight gain up to the 2^nd^ trimester and gestational weight gain, were calculated using weight measurements in the 1^st^ trimester (7–14 weeks), 2^nd^ trimester (20–28 weeks), and at delivery, which were collected from the medical records. Weight change was calculated by subtracting the self-reported pre-pregnancy weight from these values and categorizing them by proportion to pre-pregnancy weight, with cutoffs created based on both interpretability and distribution of the data (> + 3%, > 0 to + 3%, > -3 to 0%, > -5 to -3%, ≤ -5%).

The severity of NVP symptoms was assessed by a single question in the 2^nd^ trimester questionnaire: “Did you have morning sickness from conception to week 12 of your pregnancy?” (no nausea = 1, only nausea = 2, vomiting but able to eat = 3, vomiting and unable to eat = 4). We categorized other maternal socio-demographic data from the responses to questions (which was also included in the second questionnaire) as follows: annual household income (< 2 million yen, 2 to < 4 million yen, 4 to < 6 million yen, 6 to < 8 million yen, ≥ 8 million yen, no answer), maternal education (university graduate or higher, 2-year college, vocational school, high school or less), smoking status (never smoked, previously smoked but stopped before pregnancy, previously smoked but stopped because of pregnancy, current smoker). Pre-pregnancy BMI was calculated from height and pre-pregnancy weight and categorized as under 18.5 kg/m^2^, 18.5 to < 25 kg/m^2^, and 25 kg/m^2^ or higher.

Data on maternal age, parity, and birth outcomes (gestational age, birth weight, and placental weight) were obtained from medical records. We categorized maternal age and parity as follows: maternal age (< 25, 25–34, 35 ≤) and parity (0 and 1 or more). We defined small for gestational age (SGA) as a birth weight below the 10^th^ percentile of the normal population at each day of gestation and stratified by sex and parity using the Japanese birth weight reference [22]. The same reference was used to calculate the birth weight z-score for each infant.

### Statistical analysis

We first summarized participants’ background characteristics and assessed their association with weight change in the 1^st^ trimester. We also assessed the severity of NVP symptoms, gestational weight gain, birth outcomes, and their association with weight change in the 1^st^ trimester.

Next, we used linear and logistic regression to estimate the effect of weight change in the 1^st^ trimester as well as the severity of NVP symptoms, on birth outcomes. We created two models, one with and the other without weight gain up to the 2^nd^ trimester as a variable.

We considered maternal age, parity, education, household income, pre-pregnancy BMI, height, smoking status, and infant sex as confounders for the multivariate analysis. All variables were considered to be independent. Among the 91,313 subjects, weight at 7–14 weeks (*n* = 10,828; 11.9% of the sample), measurement timing of weight at 7–14 weeks (*n* = 9,741; 10.7%), weight at 20–28 weeks (*n* = 9,182; 10.1%), and measurement timing of weight at 20–28 weeks (*n* = 9,041; 9.9%), weight at delivery (*n* = 1,801; 2.0%), and placental weight (*n* = 3,562; 3.9%) were missing. Therefore, we used multiple imputations (30 imputations) to estimate the missing values from other background characteristics, birth characteristics, and existing weight measurements.

A sensitivity analysis limiting the sample to term deliveries was also conducted to ensure that the findings were not driven by differences in preterm deliveries. The analyses were repeated using categorization by actual changes in weight (≥ + 3 kg, + 1 to <  + 3 kg, 0 to <  + 1 kg, -2 to < 0 kg, < -2 kg) rather than by changes in proportion to pre-pregnancy weight to ensure the robustness of our findings.

All statistical analyses were conducted using the statistical software package Stata 13 (StataCorp. 2013. *Stata Statistical Software: Release 13*. College Station, TX: StataCorp LP.), and a *p*-value < 0.05 was considered statistically significant when performing hypothesis tests.

## Results

Table 1 shows the background characteristics of the study participants. Of the 91,313 women, 5,196 (5.7%) had a weight loss ≥ 5% in the 1^st^ trimester. The background characteristics according to weight change in the 1^st^ trimester are presented in Additional File 1. A higher proportion of women with weight loss ≥ 5% were observed among women with pre-pregnancy BMI ≥ 25 kg/m^2^ and women who never smoked. Current smokers and women who had continued smoking until they were aware of their pregnancy had a lower proportion of weight loss ≥ 5%.

**Table 1:** Background characteristics of study participants (91,313 singleton pregnancies)

	n	%
Maternal age, years		
< 25	10,337	11
25–34	58,927	65
35 ≤	22,049	24
Parity		
0	36,774	40
1 or more	54,539	60
Pre-pregnancy BMI		
< 18.5 kg/m^2^	14,783	16
18.5–24.9 kg/m^2^	67,217	74
25 kg/m^2^ ≤	9,313	10
Household income (per year)		
< 2 million	4,790	5
2 to < 4 million	29,386	32
4 to < 6 million	28,243	31
6 to < 8 million	13,634	15
8 million ≤	9,249	10
No answer	6,011	7
Maternal education		
High school or less	33,030	36
Vocational school	22,383	25
2-year college	16,116	18
University or higher	19,784	22
Smoking status		
Never smoked	52,819	58
Stopped before pregnancy	21,905	24
Stopped because of pregnancy	12,402	14
Current smoker	4,187	5
Infant sex		
Male	46,758	51
Female	44,555	49
Weight change from pre-pregnancy to 1^st^ trimester (7–14 weeks)		
> + 3%	24,975	27
> 0 to + 3%	29,525	32
> -3 to 0%	25,601	28
> -5 to -3%	6,018	7
≤ -5%	5,196	6
	mean	SD
Height, cm	158.1	5.4
Pre-pregnancy weight, kg	21.1	3.0

10,840 (11.9%) measurements of weight gain at 7–14 weeks were imputed based on other characteristics; numbers of participants for weight change from pre-pregnancy to 1^st^ trimester (7–14 weeks) add up to 91,315 due to rounding after multiple imputation

*BMI* Body mass index, *SD* Standard deviation

**Fig. 2:**
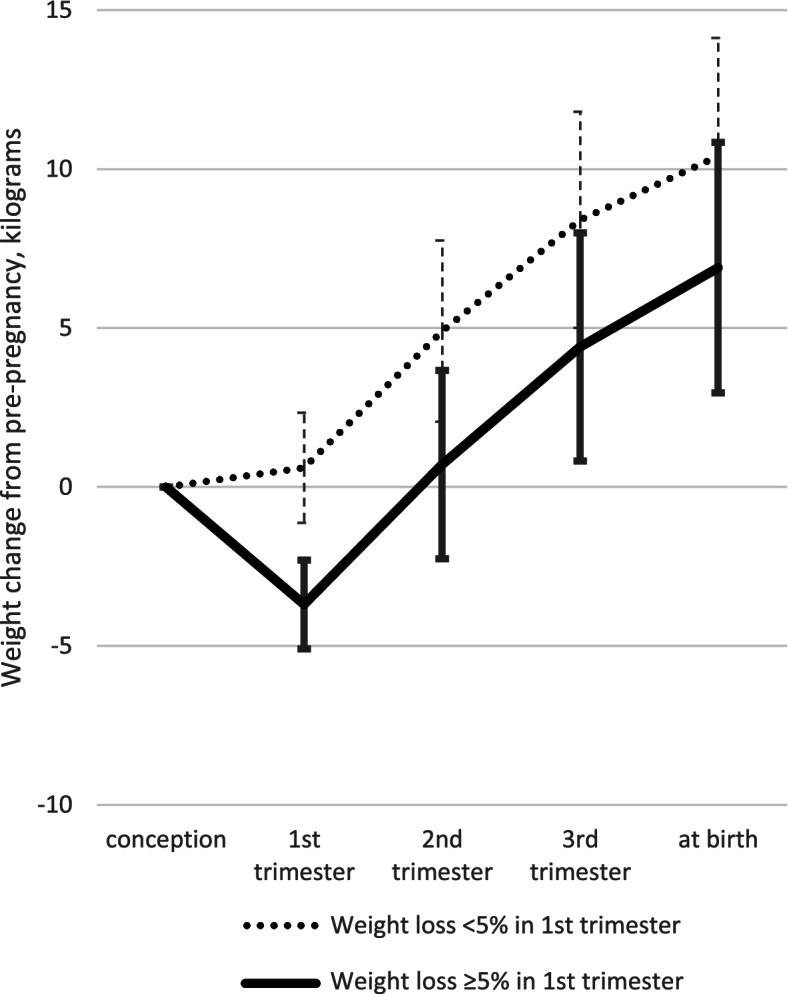
Weight gain during pregnancy among women with weight loss < 5% and ≥ 5% in 1^st^ trimester. Legends: Multiple imputation was used to impute the following missing values: weight at 7–14 weeks (*n* = 10,840; 11.9% of the sample), weight at 20–28 weeks (*n* = 9,189; 10.1%), and weight at delivery (*n* = 1,801; 2.0%)

The severity of NVP symptoms and weight change in the 1^st^ trimester were closely correlated: 73% of women who did not experience nausea gained weight, and 71% of women who vomited and were unable to eat lost weight. In the latter group, 25% had a weight loss ≥ 5%. Although more weight gain was observed in the 2^nd^ trimester among women with weight loss ≥ 5% in the 1^st^ trimester, their average weight gain was 6.6 kg less at 20–28 weeks (95% confidence interval [CI]: 6.5, 6.7), and 6.2 kg less at delivery (95% CI: 6.1, 6.3), than in women with weight gain > 3% in the 1^st^ trimester (Table 2). Weight gain up to the 2^nd^ trimester and up to delivery were all substantially lower in women with weight loss ≥ 5% in the 1^st^ trimester, although the rate of weight gain from the 1^st^ to 2^nd^ trimester was similar to that noted in women without weight loss ≥ 5% in the 1^st^ trimester (Fig. 2).

**Table 2:** Nausea and vomiting of pregnancy, gestational weight gain, and birth outcomes by weight change in 1^st^ trimester

	Weight change from pre-pregnancy to 1^st^ trimester (7 to 14 weeks)										
	> + 3%		> 0 to + 3%		> -3 to 0%		> -5 to -3%		≤ -5%		
	n	%	n	%	n	%	n	%	n	%	*p*-value +
**Severity of NVP symptoms**											
No nausea	5746	37%	5648	36%	3533	23%	427	3%	205	1%	**< 0.001**
Only nausea	11,364	29%	13,668	35%	10,955	28%	2083	5%	1196	3%	
Vomiting, but able to eat	6810	26%	8394	32%	8023	30%	1964	7%	1315	5%	
Vomiting and unable to eat	1055	11%	1815	18%	3090	31%	1544	15%	2480	25%	
	mean	SD	mean	SD	mean	SD	mean	SD	mean	SD	*p*-value +
**Gestational weight gain**											
Gestational weight gain, kg	12.8	3.8	10.4	3.3	9.2	3.4	7.9	3.7	6.6	4.3	**< 0.001**
Weight gain up to 7–14 weeks, kg	2.9	1.4	0.8	0.5	-0.6	0.5	-2.1	0.5	-4.0	1.3	**< 0.001**
―timing, weeks	11.3	1.6	11.0	1.6	10.9	1.6	11.0	1.6	11.2	1.5	**< 0.001**
Weight gain up to 20–28 weeks, kg	7.7	2.8	5.4	2.2	4.0	2.3	2.6	2.4	1.1	3.1	**< 0.001**
―timing, weeks	24.6	2.2	24.5	2.2	24.6	2.2	24.6	2.2	24.6	2.2	0.727
Weight gain from 1^st^ to 2^nd^ trimester	4.8	2.3	4.6	2.1	4.6	2.2	4.7	2.3	5.1	2.6	**0.026**
―difference in measurements, weeks	13.3	2.7	13.6	2.7	13.7	2.7	13.5	2.7	13.4	2.7	**< 0.001**
**Birth outcomes**											
Gestational age at birth, weeks	39.3	1.7	39.3	1.6	39.3	1.6	39.3	1.6	39.2	1.6	0.084
Birth weight, grams	3038	436	3032	432	3024	427	3013	413	2999	417	**< 0.001**
Birth weight z-score, SD	0.09	1.0	0.07	1.0	0.05	1.0	0.03	1.0	0.01	1.0	**< 0.001**
Placental weight, grams	561	107	558	108	558	106	554	105	553	105	**< 0.001**

The following missing values were imputed: gestational weight gain (*n* = 1,801; 2.0% of the study sample), weight gain at 7–14 weeks (*n* = 10,840; 11.9%), measurement timing at 7–14 weeks (*n* = 9,752; 10.7%), weight gain at 20–28 weeks (*n* = 9,189; 10.1%), measurement timing at 20–28 weeks (*n* = 9,048; 9.9%), and placental weight (*n* = 3,562; 3.9%)

+ : test for linear trend

Bold *p*-values: statistically significant

*NVP* Nausea and vomiting of pregnancy, *SD* Standard deviation

**Fig.3:**
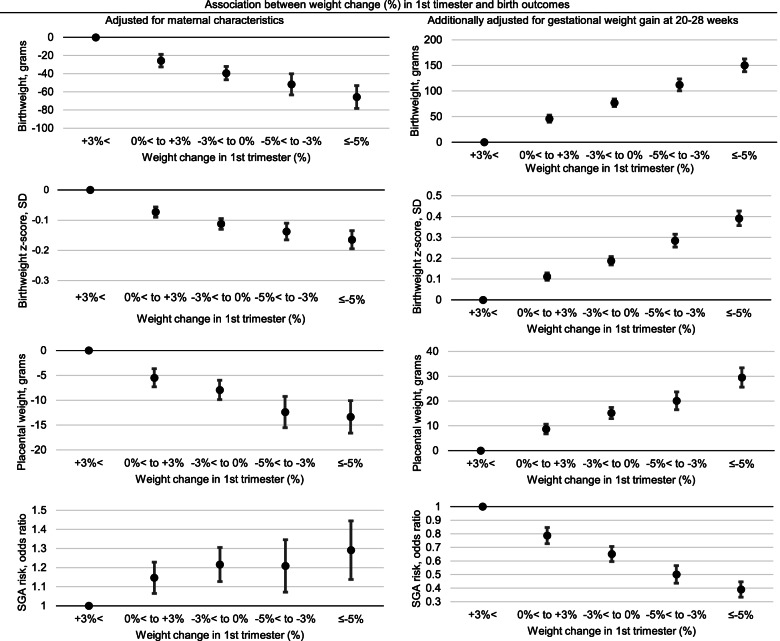
Association between weight change in 1^st^ trimester in proportion to pre-pregnancy weight and birth outcomes. Legends: Linear regressions were used for continuous outcomes (birth weight, grams; birth weight, SD; placental weight, grams), and logistic regression was used for a categorical outcome (SGA risk, odds ratio). All analyses were adjusted for maternal age, height, pre-pregnancy body mass index, household income, education, smoking status, and infant sex. Multiple imputation was used to impute the following missing values: weight at 7–14 weeks (*n* = 10,840; 11.9% of the sample), measurement timing at 7–14 weeks (*n* = 9,752; 10.7%), weight at 20–28 weeks (*n* = 9,189; 10.1%), measurement timing at 20–28 weeks (*n* = 9,048; 9.9%), weight at delivery (*n* = 1,801; 2.0%), and placental weight (*n* = 3,562; 3.9%). Weight change in the 1^st^ trimester was calculated from pre-pregnancy weight and weight at the 1^st^ trimester visit (at 7–14 weeks). Weight gain up to the 2^nd^ trimester was calculated from pre-pregnancy weight and weight at the 2^nd^ trimester visit (at 20–28 weeks). SD, standard deviation; SGA, small for gestational age

Birth weight, birth weight z-score, and placental weight were lower, and the odds of SGA were higher for women who lost more weight in the 1^st^ trimester. Women with weight loss ≥ 5% in the 1^st^ trimester had infants with lower birth weight and higher odds of SGA than women who gained weight > 3% during the same period. This association was not attenuated, instead was strengthened after adjusting for maternal characteristics. However, after adjusting for differences in weight gain up to the 2^nd^ trimester, the association was inverted; birth weight, birth weight z-score, and placental weight were increased, and the odds of SGA were decreased for women who gained less weight in the 1^st^ trimester. This made the infants from women with weight loss ≥ 5% in the 1^st^ trimester to have, on average, 150 g higher birth weight (95% CI: 135, 165) and lower odds of SGA (adjusted odds ratio [aOR]: 0.39, 95% CI: 0.33, 0.46) than those from women who gained > 3% during the same period (Fig. 3, numbers presented in Additional File 2).

**Fig. 4:**
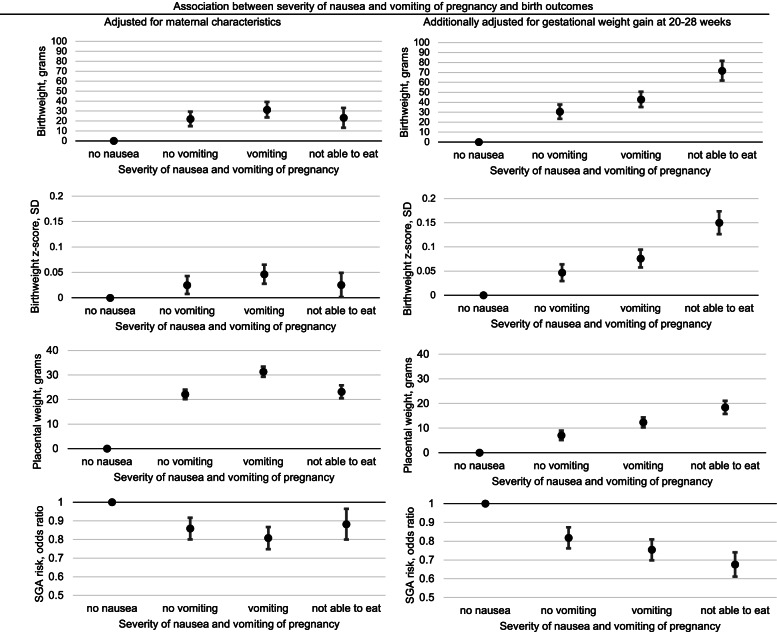
Association between severity of nausea and vomiting of pregnancy symptoms and birth outcomes. Legends: Linear regressions were used for continuous outcomes (birth weight, grams; birth weight, SD; placental weight, grams), and logistic regression was used for a categorical outcome (SGA risk, odds ratio). All analyses were adjusted for maternal age, height, pre-pregnancy body mass index, household income, education, smoking status, and infant sex. Multiple imputation was used to impute the following missing values: weight at 7–14 weeks (*n* = 10,840; 11.9% of the sample), measurement timing at 7–14 weeks (*n* = 9,752; 10.7%), weight at 20–28 weeks (*n* = 9,189; 10.1%), measurement timing at 20–28 weeks (*n* = 9,048; 9.9%), weight at delivery (*n* = 1,801; 2.0%), and placental weight (*n* = 3,562; 3.9%). Weight change in the 1^st^ trimester was calculated from pre-pregnancy weight and weight at the 1^st^ trimester visit (at 7–14 weeks). Weight gain up to the 2^nd^ trimester was calculated from pre-pregnancy weight and weight at the 2^nd^ trimester visit (at 20–28 weeks). SD, standard deviation; SGA, small for gestational age

On the other hand, women with more severe NVP symptoms tended to have larger birth weight, birth weight z-score, placental weight, and lower odds of SGA. This association was strengthened after adjusting for differences in weight gain up to the 2^nd^ trimester (Fig. 4, numbers presented in Additional File 3).

When the analysis was limited to term infants, this association persisted (Additional Files 2 and 3). A similar association was observed between weight change in the 1^st^ trimester and birth outcomes when analysis was conducted using kg measurements of weight gain instead of percentage to pre-pregnancy weight (Additional File 4).

## Discussion

This study found that women who lost more weight in the 1^st^ trimester tended to have infants with lower birth weight. On the other hand, women with more severe NVP symptoms tended to have infants with larger birth weight. When maternal weight gain up to the 2^nd^ trimester was accounted for, both women who lost more weight in the 1^st^ trimester as well as those with more severe NVP symptoms tended to have infants with larger birth weight.

There has been a controversy regarding the effects of NVP and hyperemesis gravidarum on fetal growth in previous studies [11–14]. The same kind of controversy was also found in our study results. The only difference was that our study focused on maternal weight change in the 1^st^ trimester, while previous studies included various definitions of hyperemesis gravidarum. Based on our results as well as previous studies, it can be said that NVP symptoms themselves do not have adverse effects on fetal growth, rather it is maternal weight loss, or the condition that causes maternal weight loss (e.g., malnutrition) that contributes to reduced fetal growth. This finding is consistent with recent studies that have focused on the importance of maternal weight change in early pregnancy [18, 19].

More importantly, the association between increased weight loss during the 1^st^ trimester and reduced fetal growth was inverted after adjusting for weight gain up to the 2^nd^ trimester. These results suggest that the adverse effects of maternal weight loss in the 1^st^ trimester on fetal growth may stem from the inability of those women to gain enough weight later in pregnancy to make up for their 1^st^ trimester loss, rather than from the direct influence of the severe NVP symptoms and/or weight loss in the 1^st^ trimester themselves. This decrease in total weight gain may result in malnutrition and suboptimal fetal growth. A similar argument was made by Dodds et al., who found that hyperemesis gravidarum increased the risk of SGA only if total gestational weight gain was below 7 kg, but not if weight gain exceeded 7 kg [23]. By using a larger database with longitudinal measurements of weight over the course of a pregnancy and a two-stage analytical approach adjusting for weight change persisting into mid-pregnancy, we were able to corroborate this hypothesis.

The implications of our results may potentially be significant from a preventive perspective. The adverse effects of hyperemesis gravidarum on birth outcomes, if mediated by reduced weight gain, may be reversible by increasing weight gain later in pregnancy after nausea and vomiting have resolved. This implication is relevant to the 10^th^ research priority for hyperemesis gravidarum (i.e., nutritional requirements of the first, second, and third trimesters for people with hyperemesis gravidarum), which was identified by the patient–clinician James Lind Alliance partnership [24]. Understanding the nutritional status of women with hyperemesis gravidarum, as well as effective approaches to improve it, is of great importance [25–28]. While our study has limitations inherent to its observational approach, we believe it provides insights for future interventional studies, suggesting that educational or nutritional approaches aimed at improving mid-pregnancy weight gain among women with hyperemesis gravidarum may improve birth outcomes.

The main strengths of our study include its large sample size, completeness in longitudinal measurements of weight over the course of a pregnancy, and its two-stage analytical approach hypothesizing that gestational weight gain acted as a mediator in the association between experiencing hyperemesis gravidarum and giving birth to infants with lower birth weight.

However, the limitations of the present study should be acknowledged. First, pre-pregnancy weight was self-reported and may have included a certain degree of misclassification. As such measurement error is likely to be randomly distributed across the participants, it may have led to a bias towards the null of the estimates. Second, the severity of NVP was self-rated by participants in the 2^nd^ trimester without using validated assessment tools. Detailed information related to the severity of NVP (e.g., medical interventions) was not assessed. This may have jeopardized the reliability of the data. However, the correlation between the severity of NVP symptoms and weight change in the 1^st^ trimester was high (test for linear trend: p < 0.001). Third, as an observational study, a true causal interpretation cannot be made, and the question of whether birth outcomes can be modified by weight gain later in pregnancy needs to be pursued by future intervention studies. In particular, information related to NVP symptoms after 12 weeks was not collected or assessed in the present study. We should address the possibility that NVP symptoms after 12 weeks may have caused residual confounding. In addition, each country has its own food culture, and we did not assess the diet quality or nutritional status of the participants, which may have been involved in the association between maternal weight change and birth weight. Lastly, this study was based on a cohort in Japan where women are generally thinner [29, 30], gain less weight during pregnancy [29–31], and have higher rates of hyperemesis gravidarum compared to women in other countries [1, 8, 9]. Therefore, the generalizability of our findings should be assessed in studies conducted in other populations.

## Conclusions

In conclusion, our study results suggest the possibility that the reduced fetal growth observed in pregnancies with hyperemesis gravidarum may be caused by the lack of catch-up in gestational weight gain later in pregnancy. Educational and nutritional interventions aimed at improving mid-pregnancy weight gain may improve birth outcomes in women with hyperemesis gravidarum.

## Supplementary Information

**Additional file 1.**

**Additional file 2.**

**Additional file 3.**

**Additional file 4.**

## Data Availability

The JECS data are not publicly available because of the ethical restrictions and legal framework of Japan. All inquiries about access to the data should be sent to the JECS Programme Office, National Institute for Environmental Studies (jecs-en@nies.go.jp).

## Abbreviations

aOR: Adjusted odds ratio
BMI: Body mass index
CI: Confidence interval
JECS: Japan Environment and Children’s Study
NVP: Nausea and vomiting of pregnancy
SD: Standard deviation
SGA: Small for gestational age
