# Supplementary material for: Lack of catch-up in weight gain may intermediate between pregnancies with hyperemesis gravidarum and reduced fetal growth: the Japan Environment and Children’s Study
Source: BMC Pregnancy Childbirth. 2022 Mar 12;22:199. doi: 10.1186/s12884-022-04542-0 (PMC8917715; doi:10.1186/s12884-022-04542-0)
Supplement: Supplementary file 1 — Additional file 1. [file 12884_2022_4542_MOESM1_ESM.docx]

**Additional file 1. Maternal characteristics by weight change in 1^st^ trimester**

|  |  | Weight change from pre-pregnancy to 1^st^ trimester (7 to 14 weeks) | | | | | | | | | | |
| --- | --- | --- | --- | --- | --- | --- | --- | --- | --- | --- | --- | --- |
|  |  | > +3% | | >0 to +3% | | >-3 to 0% | | >-5 to -3% | | ≤ -5% | |  |
|  |  | n | % | n | % | n | % | n | % | n | % | p-value+ |
| Maternal age, years | <25 | 3323 | 32% | 3086 | 30% | 2698 | 26% | 663 | 6% | 566 | 5% | **<0.001** |
|  | 25-34 | 15611 | 26% | 19044 | 32% | 16788 | 28% | 3998 | 7% | 3486 | 6% |  |
|  | 35≤ | 6040 | 27% | 7394 | 34% | 6114 | 28% | 1357 | 6% | 1144 | 5% |  |
| Parity | 0 | 10640 | 29% | 11628 | 32% | 9899 | 27% | 2470 | 7% | 2137 | 6% | **<0.001** |
|  | 1 or more | 14334 | 26% | 17896 | 33% | 15702 | 29% | 3548 | 7% | 3059 | 6% |  |
| Pre-pregnancy BMI | <18.5kg/m^2^ | 5189 | 35% | 4589 | 31% | 3594 | 24% | 765 | 5% | 646 | 4% | **<0.001** |
|  | 18.5-24.9 kg/m^2^ | 18150 | 27% | 22004 | 33% | 18818 | 28% | 4445 | 7% | 3801 | 6% |  |
|  | 25 kg/m^2^ ≤ | 1635 | 18% | 2931 | 31% | 3189 | 34% | 808 | 9% | 750 | 8% |  |
| Household income | <2 million | 1647 | 34% | 1399 | 29% | 1206 | 25% | 286 | 6% | 252 | 5% | **<0.001** |
| (per year) | 2 to <4 million | 8325 | 28% | 9216 | 31% | 8182 | 28% | 1929 | 7% | 1735 | 6% |  |
|  | 4 to <6 million | 7315 | 26% | 9261 | 33% | 8146 | 29% | 1901 | 7% | 1620 | 6% |  |
|  | 6 to <8 million | 3503 | 26% | 4589 | 34% | 3892 | 29% | 894 | 7% | 756 | 6% |  |
|  | 8 million ≤ | 2422 | 26% | 3226 | 35% | 2532 | 27% | 584 | 6% | 484 | 5% |  |
|  | No answer | 1762 | 29% | 1833 | 30% | 1643 | 27% | 425 | 7% | 349 | 6% |  |
| Maternal education | High school or less | 10290 | 31% | 10198 | 31% | 8750 | 26% | 2016 | 6% | 1776 | 5% | **<0.001** |
|  | Vocational school | 5764 | 26% | 7276 | 33% | 6385 | 29% | 1583 | 7% | 1375 | 6% |  |
|  | 2-year college | 4163 | 26% | 5269 | 33% | 4672 | 29% | 1099 | 7% | 914 | 6% |  |
|  | University or higher | 4758 | 24% | 6781 | 34% | 5794 | 29% | 1320 | 7% | 1131 | 6% |  |
| Smoking status | Never smoked | 12129 | 23% | 17403 | 33% | 15996 | 30% | 3931 | 7% | 3361 | 6% | **<0.001** |
|  | Stopped before pregnancy | 5849 | 27% | 7211 | 33% | 6103 | 28% | 1449 | 7% | 1293 | 6% |  |
|  | Stopped because of pregnancy | 5141 | 41% | 3679 | 30% | 2645 | 21% | 506 | 4% | 432 | 3% |  |
|  | Current smoker | 1855 | 44% | 1232 | 29% | 857 | 20% | 133 | 3% | 111 | 3% |  |
| Infant sex | Male | 13217 | 28% | 15431 | 33% | 12896 | 28% | 2896 | 6% | 2318 | 5% | **<0.001** |
|  | Female | 11757 | 26% | 14093 | 32% | 12705 | 29% | 3122 | 7% | 2878 | 6% |  |
|  |  | mean | SD | mean | SD | mean | SD | mean | SD | mean | SD | p-value+ |
| Height, cm | | 157.9 | 5.4 | 158.3 | 5.4 | 158.2 | 5.3 | 158.1 | 5.3 | 157.9 | 5.5 | **0.021** |
| Pre-pregnancy weight, kg | | 51.4 | 7.6 | 53.0 | 8.3 | 53.7 | 8.9 | 54.0 | 9.2 | 54.3 | 9.2 | **<0.001** |

10,840 (11.9%) measurements of weight gain at 7-14 weeks were imputed based on other characteristics.

+: test for linear trend

Bold p-values: statistically significant

BMI, body mass index; SD, standard deviation
