# Supplementary material for: Lack of catch-up in weight gain may intermediate between pregnancies with hyperemesis gravidarum and reduced fetal growth: the Japan Environment and Children’s Study
Source: BMC Pregnancy Childbirth. 2022 Mar 12;22:199. doi: 10.1186/s12884-022-04542-0 (PMC8917715; doi:10.1186/s12884-022-04542-0)
Supplement: Supplementary file 2 — Additional file 2. [file 12884_2022_4542_MOESM2_ESM.docx]

**Additional file 2. Association between weight change in 1^st^ trimester in proportion to pre-pregnancy weight and birth outcomes**

|  |  | Crude | | | Adjusted for maternal characteristics+ | | | Additionally adjusted for gestational weight gain at 20-28 weeks++ | | | Additionally limited to term infants | | |
| --- | --- | --- | --- | --- | --- | --- | --- | --- | --- | --- | --- | --- | --- |
| Birth weight, grams (95% CI) | | | | | | | | | | | | | |
|  | > +3% | Reference | | | Reference | | | Reference | | | Reference | | |
|  | >0 to +3% | -5 | (-12, | 2) | **-26** | **(-33,** | **-19)** | **46** | **(39,** | **53)** | **40** | **(33,** | **47)** |
|  | >-3 to 0% | **-14** | **(-21,** | **-6)** | **-39** | **(-47,** | **-32)** | **77** | **(69,** | **85)** | **67** | **(59,** | **74)** |
|  | >-5 to -3% | **-24** | **(-36,** | **-12)** | **-52** | **(-63,** | **-40)** | **112** | **(99,** | **125)** | **99** | **(87,** | **111)** |
|  | ≤ -5% | **-40** | **(-52,** | **-27)** | **-66** | **(-78,** | **-53)** | **150** | **(135,** | **165)** | **133** | **(120,** | **147)** |
| Birth weight z-score, SD (95% CI) | | | | | | | | | | | | | |
|  | > +3% | Reference | | | Reference | | | Reference | | | Reference | | |
|  | >0 to +3% | -0.02 | (-0.04, | 0.00) | **-0.07** | **(-0.09,** | **-0.06)** | **0.11** | **(0.09,** | **0.13)** | **0.11** | **(0.09,** | **0.13)** |
|  | >-3 to 0% | **-0.04** | **(-0.06,** | **-0.02)** | **-0.11** | **(-0.13,** | **-0.09)** | **0.19** | **(0.17,** | **0.21)** | **0.18** | **(0.16,** | **0.20)** |
|  | >-5 to -3% | **-0.06** | **(-0.08,** | **-0.03)** | **-0.14** | **(-0.17,** | **-0.11)** | **0.28** | **(0.25,** | **0.32)** | **0.27** | **(0.24,** | **0.31)** |
|  | ≤ -5% | **-0.08** | **(-0.11,** | **-0.05)** | **-0.16** | **(-0.19,** | **-0.13)** | **0.39** | **(0.36,** | **0.43)** | **0.39** | **(0.35,** | **0.42)** |
| Placental weight, grams (95% CI) | | | | | | | | | | | | | |
|  | > +3% | Reference | | | Reference | | | Reference | | | Reference | | |
|  | >0 to +3% | -2 | (-4, | 0) | **-5** | **(-7,** | **-4)** | **9** | **(7,** | **11)** | **8** | **(6,** | **10)** |
|  | >-3 to 0% | **-3** | **(-5,** | **-1)** | **-8** | **(-10,** | **-6)** | **15** | **(13,** | **17)** | **14** | **(12,** | **16)** |
|  | >-5 to -3% | **-7** | **(-10,** | **-4)** | **-12** | **(-16,** | **-9)** | **20** | **(17,** | **24)** | **18** | **(15,** | **22)** |
|  | ≤ -5% | **-7** | **(-11,** | **-4)** | **-13** | **(-17,** | **-10)** | **29** | **(26,** | **33)** | **27** | **(24,** | **31)** |
| SGA risk, odds ratio (95% CI) | | | | | | | | | | | | | |
|  | > +3% | Reference | | | Reference | | | Reference | | | Reference | | |
|  | >0 to +3% | 1.03 | (0.96, | 1.11) | **1.15** | **(1.07,** | **1.24)** | **0.79** | **(0.73,** | **0.85)** | **0.79** | **(0.73,** | **0.86)** |
|  | >-3 to 0% | 1.06 | (0.98, | 1.14) | **1.22** | **(1.13,** | **1.31)** | **0.65** | **(0.60,** | **0.71)** | **0.66** | **(0.60,** | **0.73)** |
|  | >-5 to -3% | 1.03 | (0.92, | 1.16) | **1.21** | **(1.07,** | **1.36)** | **0.50** | **(0.44,** | **0.57)** | **0.52** | **(0.45,** | **0.60)** |
|  | ≤ -5% | 1.09 | (0.97, | 1.24) | **1.29** | **(1.14,** | **1.47)** | **0.39** | **(0.33,** | **0.46)** | **0.39** | **(0.33,** | **0.46)** |

Multiple imputation was used to impute the following missing values: weight at 7-14 weeks (n=10,840; 11.9% of the study sample), measurement timing at 7-14 weeks (n=9,752; 10.7%), weight at 20-28 weeks (n=9,189; 10.1%), measurement timing at 20-28 weeks (n=9,048; 9.9%), weight at delivery (n=1,801; 2.0%), and placental weight (n=3,562; 3.9%)

BMI, body mass index; CI, confidence interval; SD, standard deviation; SGA, small for gestational age

+Adjusted for maternal age, height, pre-pregnancy BMI, household income, education, smoking status, and infant sex

++Adjusted for maternal age, height, pre-pregnancy BMI, household income, education, smoking status, infant sex, weight gain at 20-28 weeks (difference between pre-pregnancy weight and weight measured at 20-28 weeks [mid-pregnancy]), and the gestational age at which mid-pregnancy weight measurement was conducted

Bold values: statistically significant
