# Supplementary material for: Lack of catch-up in weight gain may intermediate between pregnancies with hyperemesis gravidarum and reduced fetal growth: the Japan Environment and Children’s Study
Source: BMC Pregnancy Childbirth. 2022 Mar 12;22:199. doi: 10.1186/s12884-022-04542-0 (PMC8917715; doi:10.1186/s12884-022-04542-0)
Supplement: Supplementary file 3 — Additional file 3. [file 12884_2022_4542_MOESM3_ESM.docx]

**Additional file 3. Association between severity of nausea and vomiting of pregnancy symptoms and birth outcomes**

|  |  | Crude | | | Adjusted for maternal characteristics+ | | | Additionally adjusted for gestational weight gain at 20-28 weeks++ | | | Additionally limited to term infants | | |
| --- | --- | --- | --- | --- | --- | --- | --- | --- | --- | --- | --- | --- | --- |
| Birth weight, grams (95% CI) | | | | | | | | | | | | | |
|  | No nausea | Reference | | | Reference | | | Reference | | | Reference | | |
|  | Only nausea | **25** | **(18** | **33)** | **22** | **(15** | **29)** | **30** | **(23** | **38)** | **21** | **(15** | **28)** |
|  | Vomiting but able to eat | **36** | **(28** | **44)** | **31** | **(24** | **39)** | **43** | **(35** | **51)** | **32** | **(25** | **39)** |
|  | Vomiting and unable to eat | **30** | **(20** | **40)** | **23** | **(13** | **33)** | **72** | **(62** | **82)** | **57** | **(48** | **66)** |
| Birth weight z-score, SD (95% CI) | | | | | | | | | | | | | |
|  | No nausea | Reference | | | Reference | | | Reference | | | Reference | | |
|  | Only nausea | **0.04** | **(0.02** | **0.06)** | **0.03** | **(0.01** | **0.04)** | **0.05** | **(0.03** | **0.06)** | **0.04** | **(0.02** | **0.06)** |
|  | Vomiting but able to eat | **0.06** | **(0.04** | **0.08)** | **0.05** | **(0.03** | **0.06)** | **0.08** | **(0.06** | **0.09)** | **0.07** | **(0.05** | **0.09)** |
|  | Vomiting and unable to eat | **0.06** | **(0.04** | **0.08)** | **0.03** | **(0.00** | **0.05)** | **0.15** | **(0.13** | **0.17)** | **0.14** | **(0.11** | **0.16)** |
| Placental weight, grams (95% CI) | | | | | | | | | | | | | |
|  | No nausea | Reference | | | Reference | | | Reference | | | Reference | | |
|  | Only nausea | **5** | **(3** | **7)** | **5** | **(3** | **7)** | **7** | **(5** | **9)** | **6** | **(4** | **8)** |
|  | Vomiting but able to eat | **11** | **(9** | **13)** | **10** | **(8** | **12)** | **12** | **(10** | **14)** | **11** | **(9** | **13)** |
|  | Vomiting and unable to eat | **10** | **(8** | **13)** | **9** | **(6** | **11)** | **18** | **(16** | **21)** | **16** | **(14** | **19)** |
| SGA risk, odds ratio (95% CI) | | | | | | | | | | | | | |
|  | No nausea | Reference | | | Reference | | | Reference | | | Reference | | |
|  | Only nausea | **0.83** | **(0.78** | **0.89)** | **0.86** | **(0.80** | **0.92)** | **0.82** | **(0.76** | **0.88)** | **0.85** | **(0.79** | **0.92)** |
|  | Vomiting but able to eat | **0.78** | **(0.72** | **0.84)** | **0.81** | **(0.75** | **0.87)** | **0.75** | **(0.70** | **0.81)** | **0.78** | **(0.72** | **0.85)** |
|  | Vomiting and unable to eat | **0.82** | **(0.75** | **0.91)** | **0.88** | **(0.80** | **0.97)** | **0.68** | **(0.61** | **0.75)** | **0.71** | **(0.64** | **0.79)** |

Multiple imputation was used to impute the following missing values: weight at 7-14 weeks (n=10,840; 11.9% of the study sample), measurement timing at 7-14 weeks (n=9,752; 10.7%), weight at 20-28 weeks (n=9,189; 10.1%), measurement timing at 20-28 weeks (n=9,048; 9.9%), weight at delivery (n=1,801; 2.0%), and placental weight (n=3,562; 3.9%)

BMI, body mass index; CI, confidence interval; SD, standard deviation; SGA, small for gestational age

+Adjusted for maternal age, height, pre-pregnancy BMI, household income, education, smoking status, and infant sex

++Adjusted for maternal age, height, pre-pregnancy BMI, household income, education, smoking status, infant sex, weight gain at 20-28 weeks (difference between pre-pregnancy weight and weight measured at 20-28 weeks [mid-pregnancy]), and the gestational age at which mid-pregnancy weight measurement was conducted

Bold values: statistically significant
