# Supplementary material for: Lack of catch-up in weight gain may intermediate between pregnancies with hyperemesis gravidarum and reduced fetal growth: the Japan Environment and Children’s Study
Source: BMC Pregnancy Childbirth. 2022 Mar 12;22:199. doi: 10.1186/s12884-022-04542-0 (PMC8917715; doi:10.1186/s12884-022-04542-0)
Supplement: Supplementary file 4 — Additional file 4. [file 12884_2022_4542_MOESM4_ESM.docx]

**Additional file 4. Association between absolute weight change in 1^st^ trimester and birth outcomes**

|  |  | Crude | | | Adjusted for maternal characteristics+ | | | Additionally adjusted for gestational weight gain at 20-28 weeks++ | | | Additionally limited to term infants | | |
| --- | --- | --- | --- | --- | --- | --- | --- | --- | --- | --- | --- | --- | --- |
| Birth weight, grams (95% CI) | | | | | | | | | | | | | |
|  | ≥ +3kg | Reference | | | Reference | | | Reference | | | Reference | | |
|  | +1 to < +3kg | **-27** | **(-37** | **-17)** | **-22** | **(-32** | **-12)** | **67** | **(57** | **78)** | **57** | **(48** | **67)** |
|  | 0 to < +1kg | **-41** | **(-50** | **-31)** | **-40** | **(-50** | **-31)** | **99** | **(88** | **110)** | **83** | **(73** | **93)** |
|  | -2 to < 0kg | **-46** | **(-57** | **-36)** | **-52** | **(-62** | **-42)** | **130** | **(118** | **142)** | **111** | **(100** | **122)** |
|  | < -2kg | **-42** | **(-55** | **-29)** | **-74** | **(-86** | **-61)** | **190** | **(173** | **206)** | **164** | **(149** | **178)** |
| Birth weight z-score, SD (95% CI) | | | | | | | | | | | | | |
|  | ≥ +3kg | Reference | | | Reference | | | Reference | | | Reference | | |
|  | +1 to < +3kg | **-0.09** | **(-0.11** | **-0.06)** | **-0.07** | **(-0.09** | **-0.05)** | **0.16** | **(0.13** | **0.18)** | **0.15** | **(0.13** | **0.18)** |
|  | 0 to < +1kg | **-0.13** | **(-0.16** | **-0.11)** | **-0.13** | **(-0.16** | **-0.11)** | **0.22** | **(0.20** | **0.25)** | **0.22** | **(0.19** | **0.24)** |
|  | -2 to < 0kg | **-0.13** | **(-0.16** | **-0.11)** | **-0.15** | **(-0.18** | **-0.13)** | **0.31** | **(0.28** | **0.34)** | **0.30** | **(0.27** | **0.33)** |
|  | < -2kg | **-0.10** | **(-0.13** | **-0.07)** | **-0.20** | **(-0.23** | **-0.17)** | **0.47** | **(0.44** | **0.51)** | **0.46** | **(0.42** | **0.50)** |
| Placental weight, grams (95% CI) | | | | | | | | | | | | | |
|  | ≥ +3kg | Reference | | | Reference | | | Reference | | | Reference | | |
|  | +1 to < +3kg | **-9** | **(-11** | **-6)** | **-5** | **(-8** | **-2)** | **12** | **(10** | **15)** | **11** | **(8** | **14)** |
|  | 0 to < +1kg | **-12** | **(-14** | **-9)** | **-9** | **(-11** | **-6)** | **18** | **(15** | **21)** | **16** | **(13** | **19)** |
|  | -2 to < 0kg | **-13** | **(-15** | **-10)** | **-11** | **(-14** | **-9)** | **24** | **(21** | **27)** | **22** | **(18** | **25)** |
|  | < -2kg | **-11** | **(-14** | **-7)** | **-17** | **(-20** | **-14)** | **35** | **(30** | **39)** | **31** | **(27** | **36)** |
| SGA risk, odds ratio (95% CI) | | | | | | | | | | | | | |
|  | ≥ +3kg | Reference | | | Reference | | | Reference | | | Reference | | |
|  | +1 to < +3kg | 1.10 | (0.99 | 1.22) | 1.10 | (0.99 | 1.22) | **0.69** | **(0.62** | **0.77)** | **0.69** | **(0.61** | **0.78)** |
|  | 0 to < +1kg | **1.23** | **(1.11** | **1.36)** | **1.27** | **(1.14** | **1.41)** | **0.60** | **(0.53** | **0.68)** | **0.61** | **(0.54** | **0.69)** |
|  | -2 to < 0kg | **1.17** | **(1.05** | **1.29)** | **1.25** | **(1.13** | **1.39)** | **0.47** | **(0.41** | **0.53)** | **0.48** | **(0.42** | **0.55)** |
|  | < -2kg | 1.10 | (0.97 | 1.25) | **1.34** | **(1.17** | **1.53)** | **0.31** | **(0.26** | **0.37)** | **0.32** | **(0.26** | **0.38)** |

Multiple imputation was used to impute the following missing values: weight at 7-14 weeks (n=10,840; 11.9% of the study sample), measurement timing at 7-14 weeks (n=9,752; 10.7%), weight at 20-28 weeks (n=9,189; 10.1%), measurement timing at 20-28 weeks (n=9,048; 9.9%), weight at delivery (n=1,801; 2.0%), and placental weight (n=3,562; 3.9%)

BMI, body mass index; CI, confidence interval; SD, standard deviation; SGA, small for gestational age

+Adjusted for maternal age, height, pre-pregnancy BMI, household income, education, smoking status, and infant sex

++Adjusted for maternal age, height, pre-pregnancy BMI, household income, education, smoking status, infant sex, weight gain at 20-28 weeks (difference between pre-pregnancy weight and weight measured at 20-28 weeks [mid-pregnancy]), and the gestational age at which mid-pregnancy weight measurement was conducted

Bold values: statistically significant
